# Experiences of disease-modifying treatments in patients with multiple sclerosis – a qualitative study

**DOI:** 10.1186/s12883-025-04576-9

**Published:** 2025-12-10

**Authors:** Kristina Gottberg, Marie Kierkegaard, Birgitta Jakobsson Larsson, Charlotte Ytterberg

**Affiliations:** 1 Department of Neurobiology, Care Sciences and Society, Karolinska Institutet, Huddinge, Sweden; 2 Academic Specialist Center, Stockholm Health Services, Stockholm, SE 113 65 Sweden; 3 Department of Public Health and Caring Sciences, Uppsala University, Uppsala, Sweden; 4 Department of Neurology, Uppsala University hospital, Uppsala, Sweden; 5 Karolinska University Hospital, Women’s Health and Allied Health Professionals Theme, Stockholm, Sweden

**Keywords:** Disease-modifying treatment, Multiple sclerosis, Qualitative design, Interviews

## Abstract

**Background:**

There is an extensive and growing body of knowledge regarding disease-modifying treatment (DMT) in multiple sclerosis (MS) regarding their effects, impact on functioning and potential side-effects. Few studies have, however, investigated the patients’ perspectives regarding their experiences of DMT in daily life.

**Aim:**

To investigate and describe the experience of DMT in the daily lives of patients with MS.

**Methods:**

A qualitative design was applied using semi-structured interviews with eight patients with MS on different types of DMT. The interviews were analyzed using qualitative content analysis.

**Results:**

Three themes emerged from the analysis; Fulfilled expectations in the light of previous treatments, Continuous need for treatment and side effect management, and Need for personalized feedback from the MS care team.

**Conclusion:**

The patients’ described experiences and hopes for personalized treatment plans, procedures, and feedback on DMT outcome implicate the need for further development of person-centred interventions. Development of such care interventions should consider the patients’ perspectives on e.g., treatment preferences and procedures, social support, and the professionalism and expert-knowledge of MS care team professionals. The knowledge derived from this study may also inform the development of patient-reported experience- and outcome measures regarding daily life with DMT in MS.

**Supplementary Information:**

The online version contains supplementary material available at 10.1186/s12883-025-04576-9.

## Background

Multiple sclerosis (MS) is a neurological disease of the central nervous system (CNS) [1]. The pathological process is characterized by inflammation, demyelination, and axonal degeneration in the CNS [2]. Being diagnosed and living with a lifelong neurological disease such as MS impacts daily life and health substantially. The development of disease-modifying treatment (DMT) to slow disease progression regarding relapse rate and disability has increased the recent years. There are today more than ten registered drugs available with different mechanisms of action, including Anti-CD20 therapies, Interferon beta, glatiramer, Cladribine, Dimethyl fumarate, Diroximel fumarate, Natalizumab, Alemtuzumab and S1PR Modulators [3–6]. The Swedish National Board of Health and Welfare’s guidelines for the care and pharmacological treatment of MS state that health care services should provide DMT for people with relapsing-remitting MS e.g., anti-CD20 antibodies and dimethyl fumarate, interferon beta and teriflunomide, but with different priorities [4]. The choice of DMT should be individualized, considering a range of medical parameters such as clinical disease activity, safety, and patient-related aspects as recommended [5, 6].

There are different routes of administration of DMTs, such as self-administered injections and oral capsules or regular infusion treatments carried out at a specialist clinic. The side-effect profiles, known from the pharmaceutical drug trials prior to approval for clinical use, also differ between the various DMTs. It is reported that adherence to DMT is low [7–9], and reasons for changing or interrupting the DMT are reportedly perceived lack of effect and/or intolerable side-effects [7–9]. It is therefore important to monitor the care and treatment processes from the patient’s perspective in order to increase the participation of patients in their health care, and to enable health care providers to improve care in the areas where patients feel that there are gaps [10, 11].

The Swedish MS Society recommends regular follow-ups by laboratory and functional tests according to specific protocols for the various DMTs [6]. There are a few quantitative questionnaires for follow-up of DMT from the patient perspective that may be applied for clinical research and quality register purposes. One example is the Treatment Satisfaction Questionnaire (TSQ) [12], which focuses on the level of satisfaction with the DMT. There is some research highlighting advantages and disadvantages of certain DMT based on reports on the TSQ questionnaire [13]. Another example is the Symptoms Frequency Intensity and Distress questionnaire (SFID-2.0), which focuses on the patient perspective of side-effects [14, 15]. Besides information on satisfaction with DMT and experience of side-effects, in-depth and more detailed knowledge is needed on patients’ daily life with DMT, knowledge that best can be revealed by qualitative studies.

Early qualitative studies on daily life with injectable DMTs [16, 17] show that although patients experienced improved quality of life, they also had uncertainties regarding treatment choice and they wished to avoid thinking about the treatment and the threat of loss of effect [17]. Further, living with DMT was perceived to become easier with time, including taking injections independently and managing side effects [16, 17]. A few, more recent qualitative studies describe the experience of patients with MS on DMT based on the range of available newly developed DMTs [18–21]. These studies show that patients with MS on different DMTs strive for normality and control while constantly confronting the MS disease, and that routes of administration and complexity of managing the DMT impact how daily life is experienced [18–23].

The knowledge from these few qualitative studies, mostly from the USA, is difficult to translate into a Scandinavian perspective due to different health care systems. Development of evidence-based care with a focus on the priority, quality areas for follow-up care of DMT in MS requires knowledge of patient-reported experiences of daily life and care. The aim of the present study was therefore to investigate and describe the experiences of DMT in the daily lives of patients with MS in Sweden.

## Methods

In order to investigate the patients’ experiences of DMT in daily life, a qualitative, descriptive design with semi-structured interviews was applied. Qualitative content analysis according to Graneheim & Lundman was performed [24, 25].

### Sample of patients with MS and DMT

A pragmatic purposeful sample of eight patients with MS were recruited from two neurological specialist care centres in Stockholm. The patients were recruited through staff at the two centres (neurologists and MS nurses) who had daily contact with patients with MS. The patients were recruited to represent a variation in type of current DMT, form of administration and sex (Table 1).

**Table 1:** Clinical-, sociodemographic- and treatment characteristics of participants with multiple sclerosis on DMT* (*n* = 8)

Sex	
Women/men, *n*	6/2
Age, years	
Median (range)	48 (39–63)
Years since diagnosis	
Median (range)	16 (7–31)
Cohabiting	
Yes/no, *n*	7/1
Children	
Yes/no, *n*	6**/2
Work status	
Yes (fulltime)/no, *n*	4/4
Previous DMTs	
Interferon beta 1a§, Interferon beta 1b, Glatirameracetate, *n*	2
Interferon beta 1a§, Glatirameracetate, Natalizumab, *n*	1
Interferon beta 1a§§, Glatirameracetate, Natalizumab, *n*	1
Interferon beta 1a§, Interferon beta 1a§§, *n*	1
Interferon beta 1b, *n*	1
Interferon beta 1a§, *n*	1
No previous DMT, *n*	1
Present DMT	
Rituximab, *n*	3
Dimethylfumarate, *n*	1
Fingolimod, *n*	1
Teriflunomide, *n*	2
Peginterferon beta-1a, *n*	1

*DMT, disease-modifying treatment. **Children at home *n* = 2

§Product A. §§ Product B

### Data collection and procedures

The patients were informed both orally and in written form about their right to withdraw at any time without the need for giving a reason. Author and interviewer KG was not involved in the participants’ care and had thus no professional relationship with them that potentially could impact the interviews. The semi-structured interviews were conducted at a place chosen by the participants, primarily at one of the two MS care centres or in the homes of the participants. An interview guide with open questions about daily life and DMT was developed and used, including questions about experiences of health care regarding treatment (available as supplementary file). The introducing questions were, “Please describe your experience of initiating your current DMT?” and “Please describe an ordinary day when you administer a dose of DMT/having a dose of DMT?”. The interviews lasted from 20 to 54 min (mean time 42 min).

### Analysis

The interviews were transcribed verbatim and read through several times. Meaning units were then identified, condensed, and analysed for their manifest and latent content. The author (KG) analysed the text from all interviews, and frequently discussed questions and uncertainties with author CY in all steps taken within the analysis. The author (MK) took part in the latter steps of the analysis. The analysis was a reciprocal process from part to wholeness and back to part from wholeness. The themes and sub-themes were refined in several meetings to address trustworthiness of the analysis. All steps taken in the analysis process is described in Table 2.

**Table 2:** The content analysis process of the patients’ experiences of DMT

Step 1. The transcribed interviews were read through in their entirety several times in order to get a sense of their content.
Step 2. The text was divided into meaning units, which consisted of the words and sentences that were related to each other through their content.
Step 3. The meaning units were condensed into shorter text that still contained the core of the meaning units and without interpretation.
Step 4. Each condensed meaning unit was labelled with codes, in a process of abstraction interpreting the underlying meaning.
Step 5. All codes were then compared with each other, and those with similar content were generated into sub-themes. The initial number of sub-themes was then reduced through a process of reading the codes, including the interpretation of the underlying meaning, and considering the whole context, thereby moving between the whole and the parts of the texts.
Step 6. The sub-themes were analysed according to their latent content, answering the question of *how *the patients experienced daily life with DMT, and three themes were created based on the underlying meaning of the sub-themes.

### Ethical considerations

The Regional Ethical Review Board in Stockholm approved the study (2017/937 − 31).

## Results

**Fig. 1:**
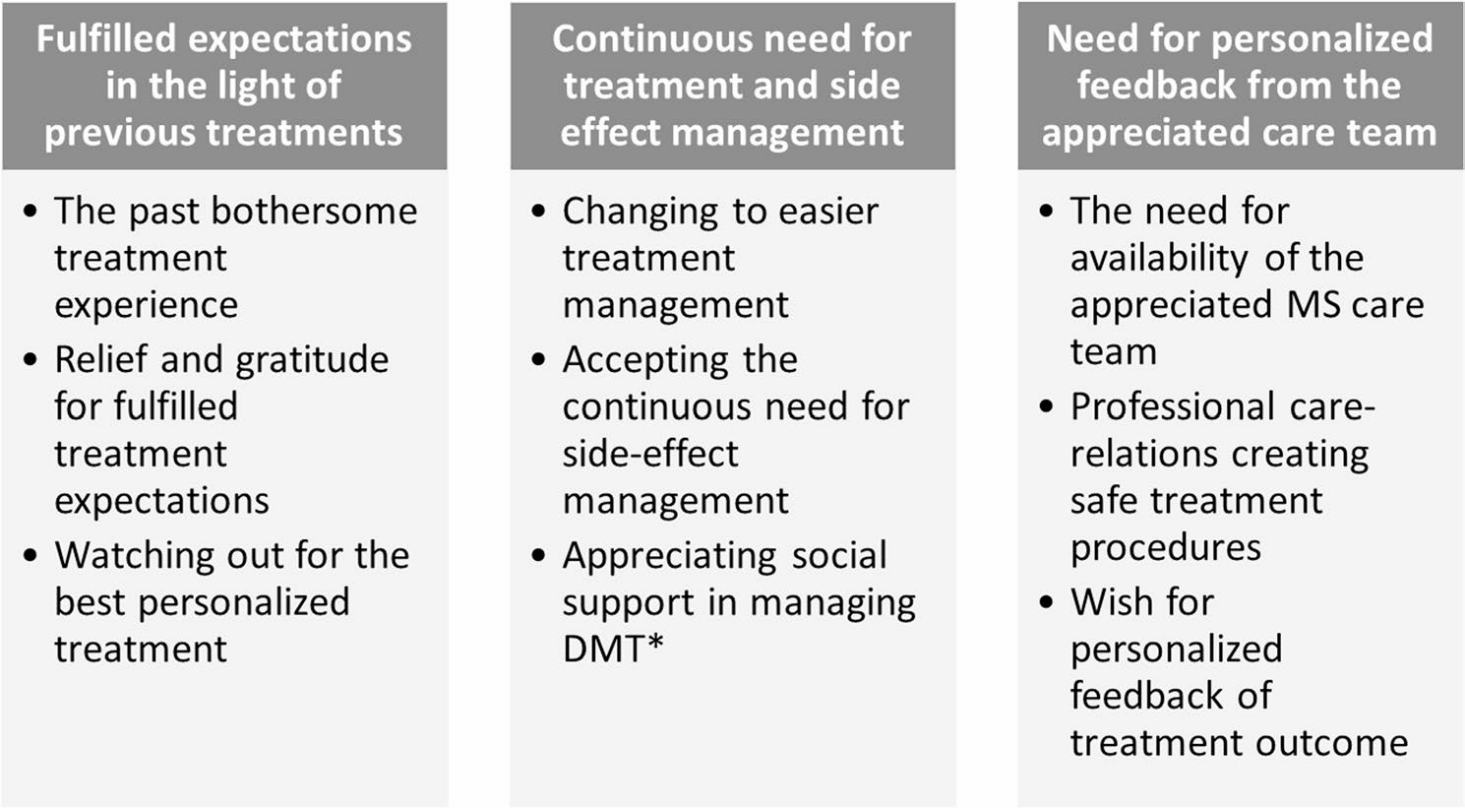
The themes and subthemes emerging from the analysis of the patients’ experiences of DMTs* in their daily lives. *DMT, disease-modifying treatment.

Three different themes emerged from the analysis describing the experiences of patients with MS regarding their daily life with DMT. The themes were: Fulfilled expectations in the light of previous treatments, Continuous need for treatment and side effect management, and Need for personalized feedback from the MS care team. An overview of themes and subthemes is presented in Fig. 1.

### Theme 1: fulfilled expectations in the light of previous treatments

#### The past bothersome treatment experience

Many of the participants had years of experience with several types of DMT prior to their current treatment. The participants described a long-winded timeline of many different DMTs, changes mixed with periods of time with no treatment at all over the recent years. Their experiences were described as bothersome and that several aspects related to DMT affected their daily life negatively. The reasons for changing or stopping their DMT several times were multi-faceted e.g., continuous ongoing relapses despite treatment or bothersome injection-related pain and local skin reactions.

*“The thing that made me stop it was that*,* in addition to having a major relapse*,* I didn’t really trust in it. After ten years you have stuck a needle into yourself everywhere and you get small lumps here and there*,* and it hurts. In the summer*,* when your skin gets a little tanned it is embarassing.” (Participant no. 4).*

Some participants described their previous DMT as controlling their daily life in terms of forced adaptations of daily activities or being dependent on informal or professional caregivers in managing the DMT. They described lack of motivation and feelings of hopelessness and being aware of their MS, with the constant need for planning when managing their DMT.

*“I thought it was really bothersome. With the Betaferon it was like every second day and the thing is I was quite careless at times with taking it properly in the end*,* the injections*,* because it was so much. I had understood that you had to be really punctual*,* that it should be the exact same time every other day*,* my life became ruled by it.” (Participant no. 2).*

#### Relief and gratitude for fulfilled treatment expectations

When comparing their newest treatment with their previous ones, several of the participants expressed that they experienced an arrest of disease deterioration, i.e., a break from severe relapses, or no relapses after their latest change of treatment. They described feelings of gratefulness, relief, and satisfaction with their improved situation. Some of the participants described that they were feeling well or even functioning better despite their treatment not being free from problems.

*You know*,* you feel fantastic*,* almost. Just*,* you know*,* feeling like it has halted for a while*,* and that I can feel that*,* that it isn’t just there – it makes you glad in a way.” (Participant no.2).*

The participants expressed gratitude with aspects such as needing less health care contacts and that their current treatment was easier to manage in daily life. A few participants described that their cognitive function had improved and that they were more alert and had more energy. On the other hand, several participants described that they were not experiencing any tangible treatment effects on a daily basis. Some participants also described that it was sometimes difficult to sort out whether their current health status was related to MS, DMT, or other health conditions and major life events.

Expectations of the treatment, i.e., to avoid severe relapses were in general high. Several participants described that their expectations were fulfilled, and their motivation to continue treatment was therefore high.

*“From my side the expectations were pretty high. I thought that*,* oh you know*,* that this might – not make me well exactly*,* not cure me completely*,* but that it would mean it wouldn’t get any worse*,* and there might not be any more relapses. On the other hand*,* I don’t think that I have had any relapses since*,* after I started with the medication.” (Participant no.5).*

In the light of their experiences from previous treatments, the participants described their current life situation with DMT as a relief, a sense of being rescued. These positive feelings of satisfaction and gratefulness originated from feeling less negative impact of their new treatment on daily life, of their lives being less controlled.

#### Watching out for the best personalized treatment

The participants described thoughts about their choice of DMT and reasoning about having or not having DMT, the type of drug and form of administration. They were always open for discussion about new possible treatments but were otherwise committed to adhere to their current treatment plan. The participants were eager to have the best possible DMT available, and were trusting their neurologist to choose the right drug. On the other hand, they feared future possible changes in DMT related to potential severe side effects or further worsening of their MS. The reasoning for treatment choice was further characterized by thoughts such as not needing the strongest drug yet, wanting to have a drug suitable for patients with milder disease severity.

*“I knew that there were tablets I was interested to get*,* but when this came along I thought we could try it. I wanted to have the mildest form because I don’t need a stronger medication yet anyway.” (Participant no.8).*

Furthermore, the participants related to their DMT as feeling safe knowing about all other possible and available treatment options, but at the same time needed to avoid thinking about MS and treatments. One participant stated that her strategy before the start of the new DMT was to avoid reading too much about the DMT to be able to dare to start the treatment. In thinking about daily life with DMT in the future, the participants expressed a wish for fast development of the DMT research field for patients with MS. They described hopeful thoughts, both for being able to continue treatment and hope for being able to change to oral DMT.

*“I mean*,* when they have come this far it is certain things will advance even further*,* so that in the end you might be able to take one tablet a week or similar*,* surely.” (Participant no. 3).*

Furthermore, some participants described fears and worries, i.e., that their treatment would be discontinued for different reasons, such as costs. One participant even described fears about moving to a new place or city where DMT would not be offered. In addition, some concerns about future, long-term negative effects of the treatment were also described by some participants.

### Theme 2: continuous need for treatment and side effect management

#### Changing to easier treatment management

The participants described their experiences of routines in daily life with their previous DMT. Several participants experienced an impact on work, for instance needing to take time off from work for treatment occasions. There were also experiences of practical difficulties with syringes and need for more support from health care staff in injection technique and managing injection-related fears. Other participants, however, described their previous experiences as having good adherence to injection routines, feeling that they were practical and simple to manage in daily life.

In comparison to previous experiences, the participants described that their current DMT procedure was easier to manage, independently of what type of administration they had changed to. It was described as a positive change with more satisfaction, e.g., a planned structure for the treatment and a limited impact on work life. For some participants, the positive experiences of their latest change of DMT were related to the form of drug administration (i.e., frequency of injections or infusions, or change to oral treatment).

*“I have of course been on the infusion drip here*,* and then you need to take a half-day off*,* several times a month. It was once this month…but it was the time – if it could be a time between one and three*,* or something of the kind*,* a bit flexible. Then I anyway still needed to take time off work.” (Participant no. 3).*

Their current routines included both strategies to not forget drug doses and other daily life strategies such as avoiding transmission of infections from other people soon after a treatment dose. Further, they planned daily life, including work life, travelling, and practical management of home treatment (for example using injection pens) to better integrate DMT in daily life. The participants’ current treatment routines were thus described as more integrated into daily life, without thoughts focusing on MS.

*“I get up in the morning and take my Aubagio and Levaxin. Then I eat breakfast and go – well*,* when I worked I went to work. I find I don’t think much about it*,* I don’t think much at all about having MS. It is just a routine like any other routine.” (Participant no. 7).*

The participants described their routines for preparation of treatment initiation in terms of practical procedures, but also preparing by gaining knowledge about the DMT. Regarding knowledge preparation, some participants described that they strategically did not prepare too much, while others described that they actively prepared and learned about their new DMT by asking the staff questions, written booklets, and some self-research on the internet.

#### Accepting the continuous need for side-effect management

The participants described their experiences of side-effects from previous treatments as more troublesome and impacting daily life more severely, in comparison to their current treatment. In some cases, the side-effects in combination with frequent relapses caused the decision to stop and change treatment. Severe skin reactions causing pain, fever and chills causing sleep problems, as well as impaired vision in relation to treatment doses were examples of side-effects and consequences that were described by the participants as negative memories.

*“…and then I got side-effects from it. It were like these small swellings and almost a feeling of having a fever etc. So it was then I stopped taking it.” (Participant no. 6).*

The participants described experiences of their current side-effects that were present during the initiation process of their treatment. They experienced relief from the process of improvement, with gradually milder or eventually disappearing side-effects. The participants experienced their current, persisting side-effects most often as mild, or less frequent, but still as a reminder of being on DMT. Some side-effects, however, were vague and it was unclear whether they were side-effects or were unrelated to the treatment, i.e., an increase in number of infections and increased fatigue. Visible side-effects such as flushing, or other skin reactions were sometimes described as embarrassing.

*“Yes*,* I can sometimes feel that I am getting hot and it is called something when you become red. Sluss-fluss… …flush! Is that the name? I get so red and look like I’m on fire*,* and I get like that sometimes. Sometimes it can be hours before it happens*,* so that sometimes I am out at the shops and then all of a sudden I turn beetroot red and everyone stares at me*,* because I look as if I am almost burning*,* and sometimes it is over my whole body too. Then the warmth comes and it can be really*,* really hot.” (Participant no. 1).*

Several side-effects were experienced by the participants as a nuisance, however, and still impacting daily life, with some of these being persistent for many years. Experience of additional fatigue and malaise in relation to treatment occasions were described by the participants as continuously affecting different aspects of daily life.

*“…I don’t really remember how long*,* but for a while*,* quite a long time*,* my stomach was affected by it. It was food in and then it was out again*,* and then food in and out (laughs). So I lost around three or four kilos or so. I was like that for a long time and my stomach – I can’t say exactly how long for*,* but it continued to play up.” (Participant no. 7)*.

The participants described stress and fear in relation to certain side-effects, such as hair loss, and side-effects occurring during the infusion treatment, such as itching in the throat. These feelings could sometimes be alleviated by an explanation from the health care professionals, thereby reducing feelings of anxiety, fear and stress.

*“I get this feeling in my mouth*,* a feeling like it is starting to itch in my throat. You get a cortisone tablet and so on before*,* and I have understood it is to alleviate this type of allergic feeling.” (Participant no.2).*

The participants’ strategies for managing side-effects in daily life were for example to cover skin reactions from injections when sunbathing, applying a pharmacological, preventive regimen, and trying to avoid troublesome situations (e.g., stomach problems). In addition, seeking help from the MS team and discussing their side effects and specific management was applied to a certain extent.

The participants described acceptance of their side-effects and not being afraid of them. They managed them independently, based on experience from previous treatments and careful preparation. In contrast, some participants described that they avoided reading and gaining too much knowledge about possible side-effects in a deliberate way, which sometimes caused more troublesome experiences of the side-effects.

#### Appreciating social support in managing DMT

Regarding support from their partners and families in DMD procedures and routines, this was typically characterized as practical issues, such as reminders to take the dose or involving children actively in self-administration of injections or having an accompanying partner for the treatment occasions at hospital.

*“Of course they have been curious*,* and I also have a daughter who is afraid of injections*,* so I asked her if she would like to come with me – and she wanted to*,* although there isn’t really any proper syringe injection for her to see. So it is more about*,* “Does it hurt?” and*,* “I can feel the needle prick but it doesn’t hurt.” So sometimes they can be there and watch…” (Participant no. 8).*

The participants described benefits from being able to share their experiences of DMT in social media patient groups and support groups. They shared experience of practical issues about DMT and also comforted each other, for example in cases of nervousness about side-effects or discussing risk for infections during treatment. This possibility to support each other was also described as valid during treatment occasions, i.e., while receiving infusions at the specialist centre.

*“There was a girl who was there for her first treatment at the same time I was there*,* and she was so nervous. I could see myself in her when I was in the same situation and had felt the same*,* so I explained to her that you just have to put up with the itching in your throat*,* because it does and I was really afraid the first time*,* although I knew that it might happen. Then she was prepared and she thanked me a lot for telling her*,* as they hadn’t said anything to her about that.” (Participant no. 2).*

The experience of relating to MS and DMT together with other patients was another important aspect described by the participants. There was a feeling of familiarity in recognizing staff and other patients during treatment occasions. This was however described as a two-sided coin, where the positive side was sharing experience, i.e., understanding weird symptoms and going on rehabilitation stays together. The other, more negative side of the coin was described as the drawback of witnessing other patients deteriorating as their disease progressed.

*“For me it’s positive. I think that*,* and it is very important to me. Above all I thought*,* I don’t think it makes a difference*,* that’s how things are. Really it is about seeing yourself in another person in the same situation (the disease and its treatment). You can understand theoretically if you read about it and such like*,* but the emotions I think you can only understand if you have been in the same position yourself.” (Participant no. 2).*

### Theme 3: need for personalized feedback from the MS care team

#### The need for availability of the attending MS care team

In the light of the usual long-term availability of regular contact with MS nurses for DMT-related questions and discussions, some participants described grief over nurses who had quit and felt insecure in not knowing what to expect in terms of availability for advice when needed. Other situations where the participants lacked availability of the expertise they needed were times of relapse and in relation to referrals to caregivers outside the MS team, for example referral to the primary care centre. Some participants experienced a need for more availability and feeling of security, expressing a need for direct telephone contact to reduce anxiety related to their MS and DMT.

*“…you want to have a contact person*,* you know*,* if something happens I need to know I can ring 24/7 and get support*,* that I don’t feel good now*,* I am worried that this has happened and just want someone to calm me down.” (Participant no. 7).*

Most of the participants, however, described satisfaction with the availability of their MS care team, resulting in a secure feeling and a sense of being taken care of. Furthermore, they described a feeling of being lucky to have the possibility of regular care contacts. They described great satisfaction with a range of external care units and systems related to their MS and DMT, such as the availability of a support group for newly diagnosed persons.

*“…if I need to ask anything I only need to call there*,* and they ring me back. So you do always have someone you can talk to*,* a MS nurse if there is anything you need to ask.” (Participant no. 1).*

#### Professional care-relations creating safe treatment procedures

Meeting health care professionals within the MS care team was experienced as being of great value. Participants much appreciated the caring relationships, e.g., with neurologists and MS nurses. Participants described that the professionals were able to assess and identify their personal needs and acknowledge the need to explain and confirm advanced aspects of DMT in scheduled visits at the specialist clinics. Furthermore, the participants valued the professionals’ ability to adapt the meeting and conversation with great perception and sensitivity to their personal needs.

*“I kind of find it difficult to concentrate now and then*,* but yes*,* that they*,* but they*,* that I – I do feel that they are skilled. It maybe isn’t such a big deal in itself*,* but they see the situation…they pick up on this and can discuss it or explain things to me.” (Participant no. 7).*

The participants experienced that the MS care team was supporting them with time and effort to maintain a hopeful attitude towards their daily life situation with DMT. The maintenance of hope was partly dependent on continuity, of meeting the same nurse and neurologist over a longer duration, i.e., many years. There were different experiences among the participants regarding sense of involvement and participation in treatment decisions or treatment changes. Some participants perceived that they took informed decisions about changing DMT in consultation with their neurologist, while others experienced that they merely followed advice and lacked a sense of participation.

*“I was of course a carrier of this JC-virus too and then that wasn’t – that was quite common as I understand it. Then there was no reason that I should risk it after a year when they didn’t think I needed it… …So I haven’t – I haven’t really been involved in any medical – so*,* and chosen without…” (Participant no. 3).*

The overall experience of participants regarding treatment situations at the specialist clinics was that they felt safe, regardless of the type of DMT, having an infusion or having support and education related to their first self-injection. Those participants who currently had DMT managed at the specialist clinics by nurses described that they were undergoing a procedure where they felt relaxed and safe in knowing they were under supervision and control. The treatment occasions were described as calm and free from stress and that they meanwhile were answering a lot of questions (verbally and digitally) related to their health. Furthermore, the participants experienced that the nurses were skilled in injection technique, which was important in decreasing anxiety.

*“The nurses are often very skilled*,* you usually hardly even get any bruising on your arm afterwards. If you are frightened of injections or needles or anything like that*,* then you don’t need to worry about that I can tell you.” (Participant no. 5).*

The participants described their gratefulness for the calm and safe treatment situation. This became evident in the light of other occasional situations when ordinary staff were not available. These occasions were far more stressful, and the participants perceived that the staff were not as knowledgeable, e.g., in listening about important treatment aspects in the treatment procedure.

#### Wish for personalized feedback of treatment outcome

Communicating and educating about DMT and feedback of follow up-results and progression of the disease were described by the participants as varying in quality. The participants experienced a need for more frequent follow-up procedures with the possibility to discuss and have treatment and disease-progression explained in more detail. They expressed a wish for more systematic feedback of test results and their treatment status. Furthermore, they reflected upon causes for the lack of information on follow-up, sometimes only receiving written letters with results from tests, i.e., after having had a magnetic resonance investigation. When little feedback was coming from the MS team, this caused further questions and reflections on the reasons for this.

*“…there wasn’t really any follow-up*,* it was just the MRI camera and then there was a letter saying things looked fine. That has been the same even in periods when I have felt wobbly. Sometimes*,* when I have been in the middle of a health dip and it has been just when I have been for the MRI*,* there has still come a letter saying*,* “Things look great*,*” you know. So then you start to wonder if your health issues are caused by something else*,* maybe it isn’t the MS.” (Participant no.4).*

One participant explained that when he did not hear from the clinic he felt a sense of being forgotten, and that there was a need to actively ask for feedback about treatment results in some way. Another participant described a negative experience receiving a letter with information about a severe side-effect (test result) that was frightening, resulting in a serious misunderstanding.

*“It was worded in such a way that I didn’t understand what it meant. When I looked it up myself it said*,* “Within three months you will be gone.” Then I was – and at the same time I couldn’t get a hold of anyone at the clinic*,* or a doctor or so on.” (Participant no. 3).*

The participants expressed that they preferred information and education about their disease and treatment face-to-face, with explanations and discussions during visits to their neurologist, rather than by written lists of side-effects or symptoms. Some participants described that some follow up-questions were relevant for other patients, not for themselves, such as questions about walking problems or hygiene-related issues in follow up of their DMT. On the other hand, they described their understanding of the relevance of performing functional tests and filling in questionnaires once a year.

## Discussion

In this qualitative interview study, we investigated the experiences of patients with MS regarding their daily life with DMT. Three themes emerged from the qualitative content analysis. The participants described that they experienced fulfilled treatment expectations in the light of their previous and more bothersome treatments, but that they still continually needed treatment and side-effect management. Furthermore, they described the need for more personalized feedback from their care team regarding DMT procedures and outcomes.

That the patients described relief after changing to easier DMT management and gratitude regarding their current DMT is not surprising in the light of their previous years with a range of more bothersome DMTs. Similarly to a study by Sippel et al. [18], most participants had experience of one to three previous DMTs before their present treatment at the time of the study. Compared with previous research with qualitative designs [20–22], participants in the present study described experiencing less impact from side effects and relief from previous, more intrusive impact from DMT in their daily lives. Whether the improved overall experience of DMT in daily life is a result of informed decisions (i.e., form of administration and treatment logistics), or acquired knowledge and experience from previous treatment years is unclear. Most likely, the sample of patients in this study had prior long-term experience that partly mirrors the development of more successful and highly active DMTs with regard to their disease-modifying effects, in combination with the patients’ personal development and adaptation to living with MS [26, 27].

The social support from peers and family members was appreciated and accepted, but seemed to be limited to practical issues and comfort in the process of giving injections or managing fears. This is in contrast to a previous study where social support from family caregivers was described as being of great importance [22]. The discrepancy might be due to differences in importance of social support regarding treatment management and that support is more important in early MS. Despite our participants’ long-term experience of MS, seeking help from the MS team for expert advice seemed to only apply to a certain extent. This highlights the importance for MS team professionals to actively ask for and monitor side effects and other DMT-related daily life aspects in a detailed way, to be able to give proper advice. MS team professionals may also advise patients to actively invite and engage family caregivers in the DMT care procedures.

Care and treatment may be a substantial part of daily life with a chronic neurological condition such as MS [26] but interestingly, to our knowledge, few studies on daily life experience with DMTs and care processes have been published [23]. In the present study, the participants were satisfied with several dimensions of care [28] related to DMT, such as experience of encounters with MS-nurses and neurologists, appreciation of their skills in DMT procedures, and their expertise and knowledge. These dimensions seemed to be important aspects of patients’ daily life experience of DMT. Similar to the study by Larsen et al. [21], this satisfaction was dependent on continuity, however, meeting the same staff in follow-up and management of DMT over long periods of time, maintaining the caring relationship developed.

Despite great appreciation for the availability of expert MS care, the participants wanted more personalized feedback. They wanted the information to be more meaningful and relevant for them regarding DMT and their daily lives. Similar results have been shown in other qualitative studies where people with MS have expressed a need for tailored and timely information in different formats (e.g., written, face-to face) [29]. From the perspective of patients, there is no direct response or effect from taking a dose of DMT that confirms the expected outcome of the DMT in slowing disease deterioration. Thus, neurologists, MS-nurses, and other professionals may consider the pedagogical task of giving feedback on process and outcome of DMT according to the patients’ individual specific needs. Furthermore, more research is needed on development of support interventions in MS specialist care regarding DMT care processes and self-management [30].

The present qualitative study has revealed more detailed knowledge on patients’ experiences of daily life with DMTs, including their experiences of care processes related to DMT management. To improve quality of care with regard to DMT management, there is a need to develop patient-reported experience measures (PREM). Patient satisfaction with care that specifically includes DMT-related aspects could form such a specific PREM. The use of patient-preferred measures for use in clinical encounters is supported by findings by Westergaard et al. [11]. The TSQ has been recommended and used in some Swedish and international studies [13, 31], but it does not incorporate aspects of DMT follow-up in clinical care, or the management of side-effects. The SFID-2.0 questionnaire [14] may guide further development of more detailed side-effect follow-up, investigating the patient-rated frequency, intensity and impact of side-effects in daily life. Some effort has been made to establish a patient-centred outcome set for MS [10], where focus group-generated data highlights the importance of the patient’s own disease experience versus clinical indicators and that measurements a specific day may not represent the long-term perspective. The results from this study and previous research [18, 20, 23] of patient experiences of DMT in daily life may thus further inform the development of person-centred feedback and DMT support, preferably in a co-design process with patients with MS and health professionals [32].

There are several methodological limitations of the present study to consider when interpreting the results. The sample of participating patients was relatively small, although the study does have a qualitative design. A larger sample might have strengthened trustworthiness and transferability of the results to other care settings. The sample of patients with MS recruited from two MS care centres in Stockholm may also represent a limited perspective, namely the perspective of patients with MS living in Sweden’s capital region, with relatively good availability of expert care. Thus, organizational differences in healthcare within and between countries may influence the transferability of the results. A strength with the present study is the qualitative, descriptive design. This facilitates a deeper understanding of the perspectives of patients with MS in Sweden regarding their daily life with DMT. The authors were experienced in conducting qualitative content analysis and were also experienced in MS care and treatment. Another strength was the purposive sampling of patients who had previously had several different types of DMTs regarding modes of administration and side-effect profiles. Further, the sample of patients showed variation in age, sex (representing the natural predominance of MS in females), and other sociodemographic factors, representing the experiences of a heterogenous population with MS. The sample of patients showed, however, less variation regarding time since diagnosis, including those with shorter disease duration.

In conclusion, the patients experienced relief when treated with their current DMT compared to previous years of bothersome treatments, learning from less successful and less implemented treatments. Continuous management and follow-up were described as needed with a person-centred and holistic approach, involving DMT management knowledge and skills, for both patients and professionals. The participants’ hopes for personalized treatment plans and feedback on DMT outcome highlight the need for further development of person-centred interventions. Development of such care interventions for initiation and follow-up of DMT should consider the patients’ perspectives on treatment preferences and procedures, social support, and expert-knowledge of MS care team professionals. The knowledge derived from this study may also inform the development of PREMs regarding daily life with DMT.

## Supplementary Information

Supplementary Material 1.

## Data Availability

No datasets were generated or analysed during the current study.

## Abbreviations

MS: Multiple sclerosis
DMT: Disease-modifying treatments
