# Supplementary material for: Experiences of disease-modifying treatments in patients with multiple sclerosis – a qualitative study
Source: BMC Neurol. 2025 Dec 10;26:31. doi: 10.1186/s12883-025-04576-9 (PMC12801913; doi:10.1186/s12883-025-04576-9)
Supplement: Supplementary file 1 — Supplementary Material 1. [file 12883_2025_4576_MOESM1_ESM.pdf]

Experiences of disease-modifying treatments in the daily lives of patients with multiple sclerosis – a qualitative study:
Interview guide for semi-structured interviews with patients on different disease-modifying treatments.

Open interview questions and probing questions

We are interested in your experiences of disease-modifying treatments, for multiple sclerosis, in your daily life.

- Please describe your experience of initiating your current DMT?
- Please describe an ordinary day when you administer a dose of your treatment/having a dose of your treatment?
    - What are your thoughts of these days and occasions?
- Since you started the treatment, how has your daily life been affected or changed in any way?
    - Please describe further.
- How do you experience taking a a dose of your treatment/having a dose of your treatment?
    - Do you experience any new symptoms or side-effects in relation to the treatment? In what way?
    - How do you manage these experiences? Are there any certain needs in these situations?
    - Were those needs met in any way? How?
- What expectations did you have on the daily life with the treatment?
    - Please describe further.
- What are your thoughts on continuation of the treatment, and the future, in your daily life?
- In relation to disease-modifying treatment in your daily life, please describe your experiences of MS specialist care?

Experiences of disease-modifying treatments in the daily lives of patients with multiple sclerosis – a qualitative study:
Interview guide for semi-structured interviews with patients on different disease-modifying treatments.

  - What are your experiences with MS specialist care and meeting health care professionals?
  - Since you started disease-modifying treatment, has MS specialist care changed in any way? In that case, please describe further?
- Please describe a situation in relation to MS specialist care and treatment that you experienced as satisfying and positive.
  - In what way? Please describe further.
- Please describe a situation in relation to MS specialist care and treatment that you experienced as less satisfying and that had potential for improvement.
  - In what way? Please describe further.
- From your experience, are there any areas for development regarding care for people with MS on disease-modifying treatment?
- What advice would you give to another person who is about to start a new disease-modifying treatment for his/her MS?

Thank you for participating!
